# Association between Betel Nut and Presence of Diverticulum in Male: A Cross-Sectional Study

**DOI:** 10.1155/2021/6669792

**Published:** 2021-04-06

**Authors:** Yu-Hong Liu, Wei-Liang Chen

**Affiliations:** ^1^Department of Surgery Medicine, Tri-Service General Hospital, School of Medicine, National Defense Medical Center, Taipei, Taiwan; ^2^Division of Family Medicine, Department of Family and Community Medicine, Tri-Service General Hospital, School of Medicine, National Defense Medical Center, Taipei, Taiwan; ^3^Division of Geriatric Medicine, Department of Family and Community Medicine, Tri-Service General Hospital, School of Medicine, National Defense Medical Center, Taipei, Taiwan; ^4^Department of Biochemistry, National Defense Medical Center, Taiwan

## Abstract

Although several studies have reported the multiple systemic effects of betel nut (BN) chewing, analyses performed on the colonic system have been few. To analyze the association between BN chewing and diverticulosis, we conducted a cross-sectional study of 5,586 eligible participants who underwent colonoscopy at a medical center in Taiwan from 2010 to 2016. BN chewing was recorded based on an assessment of personal history. Diverticulosis was categorized based on whether colonoscopies had been performed during health examinations by trained physicians at Tri-Service General Hospital. The association between different exposures, including cigarette, alcohol, BN, and diverticulosis, was also analyzed. Our study included 3,161 males and 2,425 females, and males have significantly higher prevalence rates of BN chewing than females (11.1% versus 0.3%, respectively). In the male group, BN chewing had an adjusted odd ratio (OR): 1.65(95% confident interval (CI): 1.12–2.44) with the presence of diverticulosis. Among the combination of exposures of cigarette, alcohol, and BN, the group with BN chewing combined with smoking and drinking showed significant association between diverticulosis with adjusted OR: 1.909 (95% CI, 1.188–3.065). Further subgroup analysis displayed adjusted OR: 2.310 (95% CI, 1.245–4.287) in obesity and OR: 2.406 (95% CI, 1.205–4.803) in elderly male. Thus, BN chewing is independently associated with diverticulosis in male.

## 1. Introduction

Betel nut (BN), also known as the areca nut, is the seed of the areca palm (*Areca catechu*), which grows throughout much of the tropical. Approximately 700 million people in the Pacific region, Southeast and South Asia, and parts of Africa have a habit of chewing BN, and most of them lived in Asia-Pacific regions where the *A. catechu* tree grows [1]. In fact, BN is the fourth most widely used addictive substance and major addiction in the world [2]. Studies have found various compounds in the nut, including arecoline, the main component thought to cause short-term euphoric/stimulant effects by muscarinic activity [3]. Furthermore, BN was thought to be a group 1 carcinogen in humans, and in 2004, it was linked to multiple cancers of the digestive system, such as oral cavity, pharynx, esophagus, liver, and biliary tract cancer [4]. Despite the strong link between BN and oral cancer, in recent studies, multiple systemic effects among people who chew BN were reported. In a Bangladeshi population-based prospective study, Heck et al. found that chewing BN without tobacco was associated with general hypertension (odds ratio (OR), 1.48; 95% confidence interval (CI), 1.04–2.10) [5]. In a study comprising 1,986 participants, Guh et al. presented a metabolic syndrome associated with a BN consumption rate of 10 times/day (OR, 1.31; 95% CI, 1.12–1.55) [6]. Moreover, in a population-based cohort study comprising 6,511 participants, Lan et al. found that BN chewing was associated with all-cause mortality (OR, 1.19; 95% CI, 1.05–1.35) and cerebrovascular mortality (OR, 1.66; 95% CI, 1.19–2.30) [7]. A recently emerged randomized control Chinese herbal study showed that a prescription containing one A. catechu can increase the concentrations of plasma motilin (57.69 ± 9.03 versus 49.38 ± 8.63 pg/mL, *P* < 0.01) [8].

Diverticulosis is a commonly noted status in patients who are undergoing colonoscopy with or without any gastrointestinal symptoms, and modern studies suggest that the incidence of diverticula-related disease, such as diverticulitis, is increasing [9]. A person with diverticulosis was reported to have a 10%–25% lifetime risk of suffering from it [10]. Approximately 70% of patients experience further complications, such as abscess of phlegmon, peritonitis, obstruction, and fistula, increasing the costs associated with health care [11]. Moreover, a recent retrospective cohort study demonstrated that diverticulosis posed a high risk of synchronous colorectal adenomas in patients with colorectal cancer (OR, 3.874; 95% CI, 1.843–8.144) [12]. Thus, the early detection of the presence of diverticulum might make a better prognosis for people who undergo health examinations.

To our knowledge, no research has examined the association between BN chewing and diverticulosis among the human population. Thus, we analyzed the associations between BN chewing and diverticulosis in a large general population in Taiwan using a cross-sectional study.

## 2. Methods

### 2.1. Design

**Figure 1:**
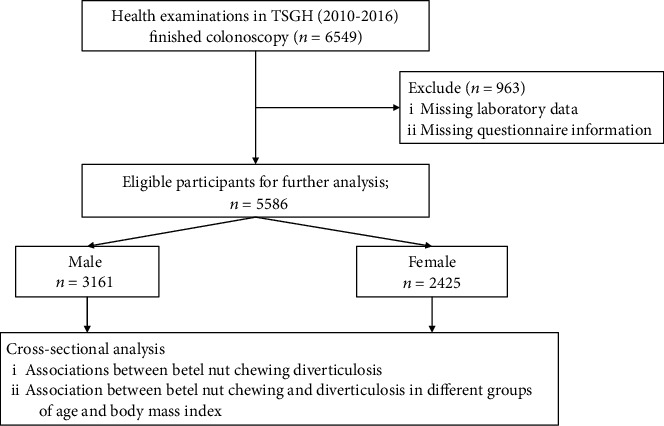
Flow chart about the steps of analysis in our study.

This retrospective cross-sectional study was investigated from health examinations in Tri-Service General Hospital from 2010 to 2016, and there were 69226 people undergoing comprehensive examinations during then. After applying the inclusion and exclusion criteria, a total of 5,586 participants were enrolled (Figure 1). Initially, we included participants who completed the informed consent while undergoing health examinations with colonoscopy. Next, we then selected participants who completed the questionnaire for personal history about substance use and excluded those who had missing elements of anthropometry measurement or serum laboratory examination. We examined the association between BN chewing and diverticulosis in different genders and then analyzed the association between BN chewing and diverticulosis in male of different ages and BMI group.

Our protocol was based on the Declaration of Helsinki and was verified by the TSGH Institutional Review Board. Before the study enrollment, all participants provided written informed consent by themselves. During the whole study, participants' characteristics were stayed anonymous, and any information about individual identification was eliminated.

### 2.2. Diagnosis of Diverticulosis

Trained physicians were responsible for performing routine colonoscopy and digital rectal examination before endoscopy examination. Those who will receive colonoscopy were told to take two doses of laxatives. The first dose was taken the night prior to examination, after which the patient began fasting. The other dose was taken on the morning of the day of colonoscopy. Participants who did not follow the protocol were told to hold off on the colonoscopy or be excluded from the study. The presence of diverticulum was recorded by the operator, and the result was categorized into two groups based on the findings of diverticulum or diverticulosis from the procedure.

### 2.3. Measurement of Covariates

**Figure 2:**
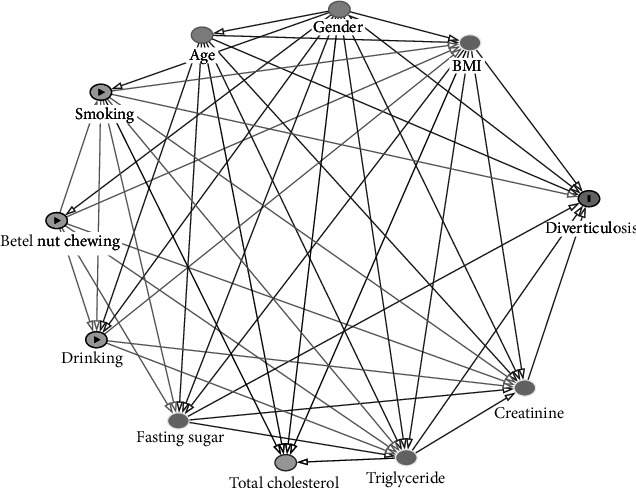
Directed acyclic graph for covariates.

Controlling confounding variables in causal inferences based on the observational data was not easy. A confounder is a factor that influences both the exposure and outcome, thus causing a spurious association. We presented our directed acyclic graph analysis of the study according to the correlations and previous evidence shown in Figure 2. Patient information, such as age, sex, cigarette smoking history, BN chewing, and alcohol consumption, were assessed using the self-report questionnaire. The question asked regarding substance use habit was “How often do you smoke cigarette, chewing BN, drinking alcohol?” The choices were “None,” “Sometimes, not every day,” or “every day.” The BMI value of a participant was derived from the body mass (weight) divided by the square of the height in units of kg/m^2^. The levels of serum total cholesterol, triglyceride, fasting glucose, and creatinine levels were assessed using standard methods. We defined the clinical obese as a BMI ≥ 27 according to Health Promotion Administration, Ministry of Health and Welfare in Taiwan [13]. We transformed the age into dichotic group at the cutoff age of 65 years old which is conventionally known as elderly.

### 2.4. Statistical Analysis

We performed Student's *t*-test and Pearson's chi-square tests to analyze the difference of laboratory data and demographic data between male and female. The associations between diverticulosis and the BN chewing were evaluated using an adjusted logistic regression model (Model 1 was unadjusted; Model 2 included Model 1 and age, serum triglyceride, serum creatinine, serum total cholesterol, serum fasting glucose, and BMI; Model 3 included Model 2 and history of alcohol consumption and cigarette use). We analyzed the present study by SPSS Inc. Released 2009. PASW Statistics for Windows, Version 18.0. Chicago: SPSS Inc., and defined statistically significant as two-sided *P* value below 0.05.

## 3. Results

The study participants were 3,161 males and 2,425 females, and their demographic and laboratory data are shown in Table 1. The mean ages were 51.67 ± 12.33 and 50.52 ± 11.82 years for males and females, respectively. Male participants had significantly higher levels of creatinine, triglyceride, fasting glucose, and body mass index than female participants. In addition, a prominently higher prevalence of alcohol drinking, BN chewing, and cigarette use was observed in the male population than in the female population.

### 3.1. Relationship between BN Chewing and Diverticulosis in Different Gender

Table 2 shows the associations between BN chewing and diverticulosis after categorizing the BN chewer by gender. A positive relationship was observed between BN chewing and diverticulosis in the male group with an OR of 1.59 (95% CI, 1.11–2.28). Moreover, significant associations were noted in Models 2 and 3 after adjusting covariates, with ORs of 1.708 (95% CI, 1.18–2.48) and 1.65 (95% CI, 1.12–2.44), respectively. However, no significant association was observed between BN chewing and diverticulosis in the female group.

### 3.2. Comparison of Odd Ratios for Diverticulosis in Male with Different Substances

To determine the different exposure effects on diverticulosis, we revealed the ORs for different combination of smoking, drinking, and BN chewing. In Table 3, small number of participants in the group of BN chewing solely (*N* = 6, 0.19%), smoking plus BN chewing (*N* = 45, 1.42%), and drinking plus BN chewing (*N* = 26, 0.82%) were noted. Among all combinations, only the group with BN chewing, smoking, and drinking simultaneously showed a significant association between diverticulosis, with an OR: 1.598 (95% CI: 1.015-2.514) and adjusted OR: 1.909 (95% CI: 1.188-3.065). The group with smoking alone (*N* = 308, 9.74%) showed OR of 0.767 (95% CI: 0.448-1.313) and adjusted OR of 0.736 (95% CI: 0.426-1.269). The group with smoking and drinking (*N* = 1019, 32.24%) showed OR of 0.994 (95% CI: 0.698-1.416) and adjusted OR of 1.0991 (95% CI: 0.759-1.569).

### 3.3. Association between BN Chewing and Diverticulosis in Males with Obesity and Old Age

We perform subgroup analysis to demonstrate the significant association between BN chewing and diverticulosis in clinical obese male and old male groups, as shown in Table 4. Because of the repetition of covariates, we deleted the BMI factor in Model 2 in the subgroup analysis for the obesity group and deleted the age factor in Model 2 in the subgroup analysis for the old age group. The statically significantly adjusted ORs in Models 1, 2, and 3 were 2.502 (95% CI, 1.418–-4.416), 2.601 (95% CI, 1.441–4.694), and 2.310 (95% CI, 1.245–4.287), respectively, in the obesity group and 2.565 (95% CI, 1.353–4.886), 2.430 (95% CI, 1.258–4.696), and 2.406 (95% CI, 1.205–4.803), respectively, in old age group.

## 4. Discussion

In our colonoscopy-based study, diverticulosis showed a significant association with BN chewing in Taiwanese male. No obvious association in the female group was found because of the fact that few participants had the habit of BN chewing, which is compatible with the low prevalence rates of BN chewing behavior among females in Taiwan [14]. Furthermore, BN chewing had a prominent effect on logistic regression among the combination analysis for most common substances: cigarettes, alcohol, and BN. To better understand how much BN chewing influences the presence of diverticulum, our subgroup analysis found that BN chewing maintained a strong link with diverticulosis based on the associated factors of being in the high-risk group, of older age, and of being clinically obese [15]. We first examined the relationship between diverticulosis and BN chewing. BN chewing, which is also known as betel quid or areca nut chewing, was thought to improve digestion and give a sensation of refreshment in users [2]. However, there is insufficient evidence of its health benefits. On the other hand, reviewing evidence of BN toxicity, the International Agency for Research on Cancer has deemed BN (with or without tobacco) as a group 1 carcinogen to humans since 2004 [4]. Furthermore, BN is not only an addictive substance; it also causes systemic effects, which are mainly caused by alkaloid arecoline-induced activation of muscarinic and acetylcholine receptors [16]. It is irrefutable that BN chewing can lead to oral disease and shows a strong association with gastrointestinal disease, as reported in recent studies. The stomach is the first stop after oral contact, and damage to the gastric system was demonstrated by Ahmed et al. in describing an increased occurrence for peptic and duodenal ulcers [17]. In addition, the liver is adversely affected by BN chewing, as was reported by Wang et al., who provided cellular evidence for the relationship between BN chewing and liver damage in rat cells [18].

Diverticulosis is a condition with the presence of colonic diverticula, and previous study showed about 4% of patient will develop acute diverticulitis in 11 years follow-up [19]. Although the clear pathological mechanisms that cause the formation of colonic diverticula are still unknown, modern studies used to believe that there were complex interactions including lifestyle, colonic dysbiosis, colonic motility, genetic factors, and microscopic inflammation [20]. However, the link between inflammation (systemic or mucosal) and the formation of asymptomatic diverticulosis was disproven by Peery et al., who demonstrated that colonic diverticulosis was not associated with mucosal inflammation [21].

There are mechanisms that link diverticulosis and BN chewing. First, BN chewing is associated with increasing gastrointestinal motility, which has been noted among patients with diverticulosis [22]. The possible mechanism for arecoline causing increased gastrointestinal motility may be the result of the stimulation of the M3 receptor at the distal colonic smooth muscle [23]. Furthermore, substances in BN stimulate the release of inflammatory mediators prostanoids, interleukin 6, tumor necrosis factor-*α* [24], and reactive oxygen species. They also activate nuclear factor-*κβ* [25], which are changes with the potential to cause chronic inflammation. The studies mentioned above could support our finding that BN chewing had higher prevalence of diverticulosis. In recent population-based study, Jarbrink-Sehgal et al. found no low-grade colonic inflammation in subjects with diverticulosis by pathologic evidence which highlighted the other possible mechanisms for the formation of diverticulosis [26]. Jones et al. also indicated no obvious alternation of gut microbiota with or without diverticulosis [27]. As evidenced by recent studies, there is an important association between colonic motility and diverticulosis, which is thought to be a factor in the strong association between BN chewing and diverticulosis presented in our study.

The present study has notable limitations. First, it has a cross-sectional design; therefore, the causal relationship between BN chewing and diverticulosis was not assessed. A long-term observation period should be considered in future studies. Second, the presence of diverticulum was observed using colonic scope and was recorded as only with the presence of diverticulum rather than further subgroup analysis for right, left, or sigmoid diverticulosis. Third, the questionnaire of daily quantity of BN use was not performed for further evaluation of the dosage effect. In addition, our study participants were not nationally represented; therefore, further study of the general population was necessary to obtain external validation.

## 5. Conclusion

In conclusion, our study reported the independently significant association between BN chewing and diverticulosis among other substance consumption or in males with obesity and old age. The patient with BN chewing should avoid smoking or alcohol drinking which would also strength the risk for diverticulosis. The effect of BN on stimulation of gastrointestinal motility might be the reason for the presence of diverticulum. Those who has BN chewing would be suggested for further evaluation of diverticulosis in order to early prevention for diverticular disease and even colonic malignancy. Further study for association between quantity of BN chewing and diverticulosis is required for more attention on this risk factor.

## Figures and Tables

**Table 1:** Characteristics of study participants of gender.

Characteristics of study participants	Gender		
	Male (*n* = 3161)	Female (*n* = 2425)	*P* value
Continuous variables^a^			
Age (years)	51.67 (12.33)	50.52 (11.82)	<0.001
Serum TC (mg/dL)	191.35 (36.82)	196.16 (37.07)	<0.001
Serum creatinine (mg/dL)	0.97 (0.32)	0.68 (0.20)	<0.001
Serum TG (mg/dL)	158.60 (99.35)	115.93 (64.64)	<0.001
Serum FG (mg/dL)	100.09 (29.61)	94.32 (21.57)	<0.001
BMI (kg/m^2^)	25.20 (3.45)	22.92 (3.64)	<0.001
Categorical variables^b^			
Smoking	1651 (52.0)	272 (11.1)	<0.001
Drinking	2091 (65.8)	712 (29.2)	<0.001
Betel nut chewing	352 (11.1)	7 (0.3)	<0.001
Diverticulosis	245 (7.7)	104 (4.3)	<0.001

^a^Values were expressed as mean (standard deviation). ^b^Values in the categorical variables were expressed as number (%). Serum TC: serum total cholesterol; Serum TG: serum triglyceride; Serum FG: serum fasting glucose; BMI: body mass index.

**Table 2:** Multivariate regression analyses for association between betel nut chewing and the presence of diverticulosis.

Models	Gender	Model^a^ 1 OR (95% CI)	*P* value	^b^ *R* ^2^	Model^a^ 2 OR (95% CI)	*P* value	^b^ *R* ^2^	Model^a^ 3 OR (95% CI)	*P* value	^b^ *R* ^2^
Variable	Diverticulosis									
Betel nut chewing	Male (*N* = 351)	1.588 (1.106-2.281)	0.012	0.004	1.708 (1.175-2.482)	0.005	0.056	1.648 (1.115-2.437)	0.012	0.057
	Female (*N* = 7)	—	0.999	0.001	—	0.999	0.131	—	0.999	0.131

^a^Adjusted covariates: Model 1 = unadjusted; Model 2 = Model 1 + age, serum TG, serum creatinine, serum TC, serum FG, BMI; Model 3 = Model 2 + history of smoking, drinking, ^b^Nagelkerke *R* squared. Serum TC: serum total cholesterol; Serum TG: serum triglyceride; Serum FG: serum fasting glucose; BMI: body mass index.

**Table 3:** The combined effects of smoking, drinking, and betel nut chewing on diverticulosis.

Smoking	Drinking	Betel nut chewing	*N* (%)	Model^a^ 1 OR (95% CI)	*P* value	Model^a^ 2 OR (95% CI)	*P* value
-	-	-	722 (22.84%)	-	-	-	-
-	-	**+**	6 (0.19%)	-	-		
**+**	-	-	308 (9.74%)	0.767 (0.448-1.313)	0.333	0.736 (0.426-1.269)	0.270
**+**	-	**+**	45 (1.42%)	0.833 (0.250-2.773)	0.766	0.806 (0.238-2.732)	0.730
-	**+**	-	761 (24.07%)	0.803 (0.540-1.193)	0.277	0.922 (0.615-1.381)	0.692
-	**+**	**+**	26 (0.82%)	1.522 (0.443-5.223)	0.505	1.741 (0.499-6.078)	0.385
**+**	**+**	-	1019 (32.24%)	0.994 (0.698-1.416)	0.973	1.091 (0.759-1.569)	0.637
**+**	**+**	**+**	274 (8.67%)	1.598 (1.015-2.514)	0.043	1.909 (1.188-3.065)	0.007

^a^Adjusted covariates: Model 1 = unadjusted; Model 2 = Model 1 + age, serum TG, serum creatinine, serum TC, serum FG, BMI. Serum TC: serum total cholesterol; Serum TG: serum triglyceride; Serum FG: serum fasting glucose; BMI: body mass index.

**Table 4:** Association between betel nut chewing and diverticulosis in male with older age and being clinically obese.

Models	Subgroup	Model 1 OR (95% CI)	*P* value	^c^ *R* ^2^	Model 2 OR (95% CI)	*P* value	^c^ *R* ^2^	Model 3 OR (95% CI)	*P* value	^c^ *R* ^2^
Variable	Diverticulosis									
Male with betel nut chewing	BMI < 27	1.186^a^ (0.729-1.930)	0.491	<0.001	1.345^a^ (0.817-2.213)	0.244	0.053	1.355^a^ (0.806-2.280)	0.252	0.053
	BMI≧27	2.502^a^ (1.418-4.416)	0.002	0.024	2.601^a^ (1.441-4.694)	0.002	0.091	2.310^a^ (1.245-4.287)	0.008	0.106
	Age < 60	1.435^b^ (0.917-2.246)	0.114	0.003	1.444^b^ (0.913-2.282)	0.116	0.047	1.402^b^ (0.869-2.263)	0.166	0.057
	Age≧60	2.565^b^ (1.353-4.866)	0.004	0.017	2.430^b^ (1.258-4.696)	0.008	0.055	2.406^b^ (1.205-4.803)	0.013	0.047

^a^Adjusted covariates: Model 1 = unadjusted; Model 2 = Model 1 + age, serum TG, serum creatinine, serum TC, serum FG; Model 3 = Model 2 + history of smoking, drinking. ^b^Adjusted covariates: Model 1 = unadjusted; Model 2 = Model 1 + BMI, serum TG, serum creatinine, serum TC, serum FG; Model 3 = Model 2 + history of smoking, drinking. ^c^Nagelkerke *R* squared. Serum TC: serum total cholesterol; Serum TG: serum triglyceride; Serum FG: serum fasting glucose.

## Data Availability

The data that support the findings of this study are not publicly available due to legal restrictions imposed by the government of Taiwan in relation to the “Personal Information Protection Act”; data cannot be made publicly available.
